# Working in the Eye of the Pandemic: Local COVID-19 Infections and Daily Employee Engagement

**DOI:** 10.3389/fpsyg.2021.654126

**Published:** 2021-07-01

**Authors:** Max Reinwald, Sophia Zimmermann, Florian Kunze

**Affiliations:** ^1^Institute for Leadership and Organization, Ludwig-Maximilians-Universität München, Munich, Germany; ^2^Chair for Organisational Studies, University of Konstanz, Konstanz, Germany

**Keywords:** COVID-19, work engagement, diary study, leadership, aging

## Abstract

The COVID-19 pandemic has drastically changed many aspects of our society and work life. This study assesses how daily variations in employees' work engagement are affected by daily variations in infection rates in employees' communities. Applying the conceptual framework of event system theory, we argue that surging COVID-19 cases have an impact on employee engagement, depending on the individual sensemaking processes of the pandemic. We assume that employee age and received leader support are key context factors for these sensemaking processes and that particularly older employees and employees who receive little leader consideration react with lower work engagement levels toward rising local COVID-19 infections in their proximity. We find support for most of our proposed relationships in an 8-day diary study of German employees, which we integrate with official COVID-19 case statistics on the county level. We discuss the implications of these results for the literature on extreme events and individual workplace behavior. Furthermore, these findings have important implications for companies and executives who are confronted with local COVID-19 outbreaks or other extreme societal events.

## Introduction

The COVID-19 pandemic has turned life upside down. The rapid worldwide spread of COVID-19 caught organizations and communities off-guard. The scale of the pandemic is reflected in the exponential increase in new cases every day, requiring organizations and communities to continuously adjust work and life routines. For example, on August 1, 2020, the World Health Organization confirmed 289,321 new COVID-19 cases over the last 24 h worldwide (World Health Organization, 2020e).

From an organizational perspective, the COVID-19 pandemic can be described as an extreme, disrupted context. Such environments are “triggered by extreme events that occur outside the core activities of organizations or communities” (Hällgren et al., 2018, p. 135). We consider the day-level fluctuation of COVID-19 cases as extreme daily events since this disease results in unbearable physical, psychological, and material consequences to organizational members.

The reach of the COVID-19 pandemic raises the question if organizational members are affected in their work behavior by the extreme event of local COVID-19 case fluctuation and how organizations, and particularly their leaders, can intervene to prevent negative spillover of the external events on their employees. The current empirical literature on extreme disrupted contexts does not provide conclusive answers to this question. Further, the disrupted context literature appears to have mainly focused on the development of temporal organizations and the role of stakeholders during disruptions (Hällgren et al., 2018), thus neglecting possible cross-level effects of extreme events on individual-level outcomes. Nonetheless, related research provides indications that acute, extra-organizational stressors, such as extreme weather or terrorist attacks, can affect individual work outcomes, e.g., absenteeism (Kivimäki et al., 1997; Byron and Peterson, 2002), burnout (Toker et al., 2015), job satisfaction, job intension, and work intensity (Hochwarter et al., 2008). Yet, the organizational behavior literature has devoted surprisingly little attention to understanding relations between extreme events, such as natural disasters or terrorism, and within-organizational consequences, considering the high frequency with which such catastrophes tend to occur (James, 2011). As a result, James (2011) calls for more empirical research on disaster and terrorism from an organizational behavior perspective.

With this study, we want to address this crucial research gap in the literature on extreme events by considering the COVID-19 pandemic context and its effects on daily employee work behavior. In particular, we focus on employee engagement, defined as “the simultaneous employment and expression of a person's ‘preferred self' in task behaviors that promote connections to work and to others, personal presence (physical, cognitive, and emotional) and active, full performances” (Kahn, 1990, p. 700). Work engagement has profound implications for employees' performance and their psychological and physical well-being (Bakker et al., 2008). Contributing to personal health and business survival, engagement is, therefore, a relevant factor to be considered in an extreme, disrupted context, such as the COVID-19 pandemic. Engagement research suggests that individuals' engagement levels fluctuate heavily across days and are influenced by within-organization negative work events (e.g., making errors, working under time pressure, conflicts; Bledow et al., 2011; Demerouti and Cropanzano, 2017). Extending this line of thought, we expect that work engagement waxes and wanes in response to outside organizational events, such as daily local COVID-19 infections.

Drawing on event system theory (Morgeson et al., 2015), we propose that employees make sense of the daily number of COVID-19 cases reported in an employee's local area. Referring to classical ideas of sensemaking processes, as the conceptual backbone of the event system theory, personal, and organizational factors play a crucial role, if individuals interpret an event as salient for sensemaking and respective behavioral changes or not (Maitlis and Christianson, 2014). We thus assume that it depends on personal factors (i.e., employees' age) and organizational factors (i.e., perceived leadership support) if employees interpret surging infection numbers as a strong event that interrupts their work engagement. Specifically, we suggest that rising infection rates are particularly harmful to the engagement of older compared to younger employees, as the COVID-19 pandemic puts especially the health of older employees at risk. Furthermore, employees should be able to cope better with increasing COVID-19 infection rates, if they have a leader who shows consideration, defined as “leadership behavior that involves concern for employees' well-being, expressions of support, and displays of warmth and approachability” (Lambert et al., 2012, p. 913). Combining both contextual factors, older employees under the supervision of someone who does not care about their specific needs will typically experience the worst effect of local COVID-19 numbers on their work engagement.

**Figure 1:**
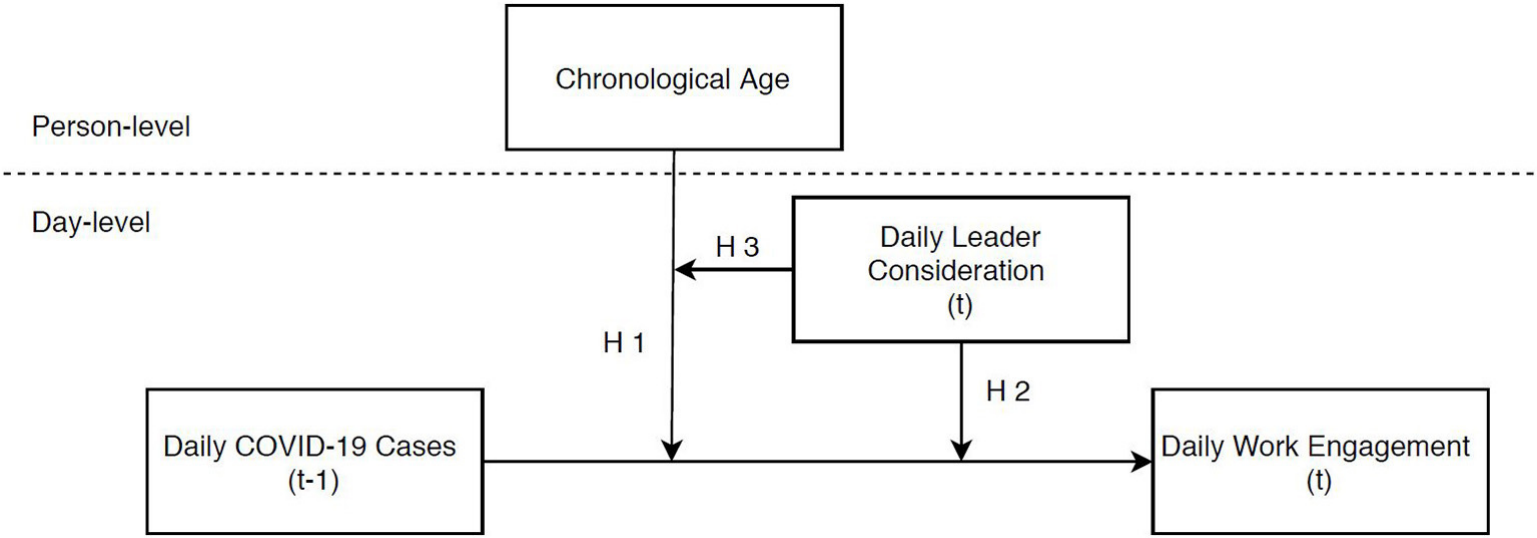
Conceptual model.

To test our theoretical model, as depicted in Figure 1, we integrated objective information on daily local COVID-19 cases in Germany with survey data collected through a diary study over eight consecutive workdays at the beginning of April 2020. In doing so, we intend to contribute to theory and practice by answering the question of when extreme daily events at the environmental level (i.e., the daily local COVID-19 surge) may spill over into the organizational workplace by affecting individual daily work behavior (i.e., daily employee engagement). Moreover, we aim to provide insights into effective leadership behaviors that may buffer the impact of extreme events on employee work behavior, and thereby help companies to master external crises, like the Covid-19 pandemic situation and other extreme situations of natural or manmade origin.

### Theory and Hypotheses Development

Event system theory (Morgeson et al., 2015) suggests that events originate at any hierarchical level—from the most molar environmental level to the most molecular individual level—and their effects can travel up or down throughout organizations. Yet, not all events will trigger changes in organizational behavior (Nigam and Ocasio, 2010), but it is the event strength reflected by the event novelty, disruption, and criticality that attracts attention and evokes behavioral change within organizations (Morgeson et al., 2015).

We propose that the daily number of local COVID-19 cases has the potential to function as a strong event as employees perceived them as novel, disruptive, and critical. Before its worldwide outbreak, the COVID-19 virus was an unknown, potentially fatal disease (World Health Organization, 2020a). *Novelty* stems from the fact that the virus suddenly emerged as a fatal disease (World Health Organization, 2020a). Therefore, the development of the infection rates was unpredictable, and most countries, such as Germany and the United States, reported new infections daily on domestic and district levels (Johns Hopkins University Medicine, 2020; Robert Koch Institut, 2020). In Germany, the novelty of the COVID-19 cases was especially striking at the beginning of April, when the number of cases skyrocketed. This exponential increase in COVID-19 cases *disrupted* work and life routines, as social distancing measures required an economic and societal shutdown, which forced employees to adjust and adapt their general and work-related behaviors (World Health Organization, 2020d,f). Finally, local COVID-19 infection rates also constitute a *critical* event through the virus's transmission mechanism. The COVID-19 virus is mainly transmitted via social contact, meaning that surging case numbers in an individual's local environment increase the risk of infection (World Health Organization, 2020b). Daily local COVID-19 cases, therefore, reflect an individual's chance to fall ill from the virus and, thus, to endure physical, psychological, and material hardship.

Yet, not all employees may perceive local infection rates as being a strong event, as another core idea of the event system theory (Morgeson et al., 2015) is that organizations and organizational members reach perceptions of the urgency of a specific event through sensemaking processes (Weick, 1988, 1995). We propose that both individual characteristics (i.e., employee age) and perceived support from a direct leader (i.e., leader consideration) matter for this sensemaking process and thus trigger variation in the effect of daily COVID-19 cases on employee engagement.

### Employee Age and the Effect of Local COVID-19 Cases on Work Engagement

Current statistics on COVID-19 indicate that the risk of experiencing severe physical symptoms and health impairments rises with chronological age (Centers for Disease Control Prevention, 2020). Adults over 50 years already have a significantly higher fatality rate than the average 2.3% reported worldwide (World Health Organization, 2020c). This pronounced illness susceptibility due to the COVID-19 pandemic throws the mortality of elderly employees into sharp relief (Kanfer et al., 2020). Not surprisingly, a representative survey in the United States showed that older individuals perceive a higher risk of dying from COVID-19 (Bruine de Bruin, 2021).

Given these health threats, aging employees may make sense of COVID-19 case numbers in their direct environment, as a particularly strong event encouraging them to adapt their work-related behavior accordingly. Being constantly reminded of their increased health risk by the daily COVID-19 surge, aging employees may not be able to drive their physical, cognitive, and emotional energies into their work-role performances as much as under normal circumstances (Rich et al., 2010). They are also somewhat likely to invest the “hands, head, and heart” (Ashforth and Humphrey, 1995, p. 110) in putting themselves out of risk. Therefore, aging employees may continuously engage in processing extensive COVID-19 information provided by the media to re-evaluate their courses of action based on the current state of knowledge of the disease. These considerations might include questions of whether, when, and how to continue working (Kanfer et al., 2020). Hence, they allocate a substantial amount of resources that they usually use to perform their work roles in making sense of the COVID-19 pandemic. Daily news of local COVID-19 cases is, therefore, likely to hold elderly employees from being “psychologically present, fully *there*, attentive […] and focused in their role performances” (Rich et al., 2010, p. 619). Research on critical events (Gersick and Hackman, 1990; Morgeson and DeRue, 2006) further substantiates this notion, indicating that significant events tend to command attention, influence resource allocation, and ultimately have implications for employee performance. Consequently, we propose that the daily number of local COVID-19 cases and employees' age interact in their relation to work engagement leading to the following hypothesis:

*Hypothesis 1:* There is a two-way interaction between the daily number of local COVID-19 cases and employees' age on employees' work engagement such that local COVID-19 cases negatively relate to employees' daily work engagement for older employees, but the relationship becomes less negative as age decreases.

### Leader Consideration and the Effect of Local COVID-19 Cases on Work Engagement

A further critical factor that affects sensemaking processes of external events, such as a pandemic gaining momentum in one's direct community, is perceived leadership behavior. Extreme contexts are environments where leadership is needed most (Hannah et al., 2009). During the COVID-19 pandemic, leaders face the challenge of leading in an extreme and volatile context (Hannah et al., 2009; Hannah and Parry, 2014). All of their followers are personally affected to some extend by the pandemic depending on the perceived strength of the event (Hannah and Parry, 2014). A strong event might occur particularly for aging employees due to the aforementioned health threats but also some of the younger or middle-aged employees confronted with increased child-care obligations (Cluver et al., 2020) or loneliness (Banerjee and Rai, 2020) may perceive a strong event.

Therefore, we argue that leaders showing a high degree of daily consideration are effective in the COVID-19 context. Considerate leaders are empathetic and competent at sensing and subsequently satisfying their followers' needs (Judge et al., 2004). Furthermore, they are distinctly concerned about their followers' health and well-being (Lambert et al., 2012). Hence, considerate leaders are likely to support their followers' continuous sensemaking efforts helping them to “get their bearings and then create fuller, more accurate views of what is happening and what their options are” (Weick, 1988, p. 310). Followers may further feel comfortable sharing their potentially debilitating emotions with their supervisors since considerate leaders typically display high levels of warmth and approachability.

Thus, experiencing daily leader consideration, employees are likely to feel supported in their sensemaking process and emotion regulation. As a result, we argue that they can better cope with the daily number of local COVID-19 cases. Therefore, we propose that daily leader consideration empowers employees to recover their previous level of work engagement. Employees might, thus, be enabled to harness their “full self in terms of physical, cognitive, and emotional energies to work role performances” (Rich et al., 2010, p. 617) despite experiencing extreme daily events. In contrast, employees who perceive little individual support from their direct leaders might view the surge in COVID-19 infections as a strong event, thus distracting them from investing full effort into their job. Consequently, we argue that daily leader consideration behavior is effective in mitigating an adverse effect of the daily number of local COVID-19 cases on daily work engagement leading to the following prediction:

*Hypothesis 2:* There is a two-way interaction between the daily number of local COVID-19 cases and leader consideration on employees' work engagement such that local COVID-19 cases negatively relate to employees' daily work engagement for employees with low leader consideration, but the relationship becomes less negative as leader consideration increases.

### Interplay Between Age and Leader Consideration and the Effect of Local COVID-19 Cases on Work Engagement

Integrating the prior arguments, we assume that, in the face of rising local COVID-19 cases, older employees react more favorably to received daily leader consideration compared to their younger colleagues. Older employees are likely to experience extreme emotions, such as terror, fear, and distress, due to the heightened mortality salience caused by rising infection rates in their environment (Janoff-Bulman and Frieze, 1983; Arndt et al., 1997). Consequently, they have the highest need for support from their supervisors for sensemaking that lowers their critical interpretation of the extreme health event. If they receive the needed consideration from their leader, they are less likely to interpret the local COVID-19 cases as a personally threatening event and might feel their leader effectively acknowledges and accommodates their needs (Lambert et al., 2012; Maitlis and Christianson, 2014). On the other hand, if they do not receive such support, their work engagement is likely to be most negatively affected among all employee groups. They might feel that their supervisor does not understand or care about their personally threatening situation and does not come to their aid (Lambert et al., 2012). Without support from their supervisor to cope with the critical health situation, they will allocate their resources away from work-related engagement, focusing all their efforts on sustaining their health.

In contrast, younger employees may feel less threatened by rising COVID-19 cases because their lower age indicates lower personal health risks. While they might generally react favorably to leader consideration, already moderate levels of consideration might suffice to match their needed consideration, and a further increase in leader consideration beyond this need may have a decreasing marginal impact on their engagement (Lambert et al., 2012). In consequence, we formulate the following final hypothesis:

*Hypothesis 3:* There is a three-way interaction among the daily number of local COVID-19 cases, employees' age, and daily leader consideration on employees' work engagement such that the buffering interaction between COVID-19 cases and leader consideration is more pronounced as employee age increases.

## Methods

### Procedure and Participants

We collected the study data during the peak of the first COVID-19 wave in Germany from March 30 through April 9 (based on official government data, April 2, with 6.561 cases, the highest number of newly reported cases in spring 2020). The sample was recruited in cooperation with a survey company (Respondi), which gave us access to its online panel of participants located all over Germany. Survey participants mirrored the German working population in terms of gender structure and age distribution, based on the most recent published data from the German statistical office (Statistisches Bundesamt, 2018). Furthermore, participants were only allowed to participate if they (a) had a working contract, and (b) were currently at least partly working from home. These requirements were ensured through a presurvey. Respondents had to complete a general survey on day 1 (Monday, March 30) and then participate in eight daily surveys over the next eight workdays. We did not survey for the full 2 weeks, as the potential ninth day (April 10) was an official holiday in Germany. The daily surveys were open from 6 p.m. through 8 a.m. the next morning. Participants received incentives of 0.75€ for the general survey, 0.25€ for each daily survey, and a bonus of 1.00€, if they participated in all eight daily surveys. The initial sample at day 1 consisted of 699 participants, who were mainly male (58%), on average 44 years old, and worked 80% of their time from home. In line with prior work (e.g., Rosen et al., 2016; Gabriel et al., 2018), we retained data for participants who provided daily data for more than three workdays to assure that the momentary assessments are representative of participant's individual experiences and are not biased toward days with extreme experiences (Ohly and Gochmann, 2017). This resulted in a final sample of 388 participants who provided 2.858 daily surveys.

### Measures

Age as a person-level variable was collected in the general survey on day 1. COVID-19 cases, engagement, and leader consideration were assessed on a daily level for eight consecutive workdays.

#### Age

The chronological age of the participants was captured in the baseline survey in years lived since birth.

#### Daily COVID-19 Cases

To capture daily local COVID-19 cases for every individual participating in our survey, we used the official data released by the Robert Koch Institute (RKI) in Germany. The RKI is the government's central scientific institution for disease control and prevention and provides the number of daily COVID-19 cases disaggregated at the county level. This allowed us to map officially confirmed COVID-19 cases in a county to each survey participant on a daily level using the postal code provided by the participants. On the last day of our survey, over 119,000 official confirmed cases culminated in 412 counties in Germany. By law, each laboratory-confirmed COVID-19 case has to be notified to the local public health department and transmitted to the RKI. The RKI visualizes the data and makes them easily accessible on a county-level in an online dashboard and daily situation reports (https://www.rki.de/DE/Content/InfAZ/N/Neuartiges_Coronavirus/Situationsberichte/Gesamt.html). Because of the easy accessibility of the data and the wide coverage of case numbers by the local and national press, most individuals are aware of local COVID-19 outbreaks. We used the absolute numbers of COVID-19 cases in a county around a participant in our analysis, as absolute numbers were the dominant metric of reporting at this time. We lagged this measure by 1 day (*t* – *1*) to assure that COVID-19 cases temporally proceed the outcome measure, which allows for stronger causal conclusions.

#### Daily Work Engagement

We assessed daily work engagement using six items from the work engagement scale by Rich et al. (2010). Following the recommendation for diary studies (Uy et al., 2010; Gabriel et al., 2019), we used a shortened version of the original scale to reduce participant fatigue and adapted the scale for day-specific assessments. Each dimension of the scale is measured with two items, for example, “Today I tried my hardest to perform well on my job” (physical engagement), “Today I was enthusiastic in my job” (emotional engagement), “Today at work, I focused a great deal of attention” (cognitive engagement). For each dimension, the two items with the highest factor leadings as reported by Rich et al. (2010) were selected. Items were rated on a five-point scale ranging from *strongly disagree* to *strongly agree*. The average coefficient alpha across the 8 days was 0.935.

#### Daily Leader Consideration

We used the three-item scale from Lambert et al. (2012) to measure leadership consideration, which does not confound leader behaviors and the outcomes of leader behaviors. The items asked participants for the following leader behaviors of their direct supervisor referencing to the current workday: “Acting friendly and approachable,” “Acting concerned for my personal welfare,” and “Acting supportive when talking to me.” As such, the items measured the amount of support received from the direct supervisor. The items were assessed on a scale ranging from 1 (*never*) to 5 (*very often*). Cronbach's alpha was 0.960 on average.

### Data Analysis

Given the nested data structure (i.e., days nested within persons), we tested all hypotheses using mixed-effect models in Stata SE 16. All day-level variables (i.e., COVD-19 cases, engagement, leader consideration) were treated as Level 1 (within-person) variables, and age as a person-level variable was treated as Level 2 variable (between-person) in the model. Because the nesting of individuals in counties is more a nuisance than of substantial interest for our hypotheses, we used clustered standard errors, which account for potential interdependence between observations from the same county. An advantage of using clustered standard errors is that they do not require additional assumptions about the appropriate specification of random effects for the county level (McNeish et al., 2017).

Following prior recommendations (Enders and Tofighi, 2007), we person-mean centered the Level 1 predictors. The person-mean centering of Level 1 variables effectively eliminates between-person variance and provides a pure estimate of day-level relationships between daily individual local exposure to COVID-19 cases and daily individual engagement as postulated in our hypotheses. Because person-mean centering of the Level 1 predictors removed all stable between-person differences (e.g., demographics, personality, response tendencies), such stable between-person differences could not bias our estimates and were not controlled for (Enders and Tofighi, 2007; Gabriel et al., 2019). However, as recommended (Singer and Willett, 2003), we controlled for time (with dummy variables), as the predictors and the outcome might systematically change throughout the study (e.g., due to adaption processes to the pandemic). We modeled all hypothesized day-level effects as random slopes and time as a fixed slope (for similar procedures, see Wang et al., 2011; Lanaj et al., 2021).

## Results

Descriptive statistics, reliabilities, and day- and person-level correlations are displayed in Table 1.

**Table 1:** Means, standard deviations, reliabilities, and correlations of study variables.

	**Variable**	**Mean**	***SD***	**1**	**2**	**3**	**4**
1	Age	44.887	11.923	—			
2	Engagement	3.999	0.655	0.150^**^	(0.935)	−0.006	0.128^**^
3	COVID	0.501	0.734	0.037	−0.057	—	0.002
4	Leader consideration	2.944	1.127	−0.162^**^	0.350^**^	−0.072	(0.960)

Correlations above the diagonal are day-level correlations (N = 2,585). Correlations below the diagonal are person-level correlations (N = 388). Day-level variables were aggregated to the between-person level prior to computing person-level correlations. Because nesting of observations in persons is not accounted for significance values for day-level correlations should be interpreted with caution. Coefficient alpha averaged across all time points are shown along the diagonal in parentheses. Means and standard deviations (SD) were computed at the person-level of analysis. COVID variable is rescaled (COVID Cases/1,000). ^*^p < 0.05 (two-tailed);

** *p < 0.01 (two-tailed)*.

Before testing our hypotheses, we first inspected the intraclass correlation coefficient (ICC1) for our criterion measure, as substantial day-level variation in outcomes is a prerequisite for examining day-level relationships using mixed-effect models (Hox, 2010; Raudenbush and Bryk, 2010). An ICC1 of 0.542 for engagement indicated that 54.2% of the variance in engagement lies between-individuals and 45.8% of variance within-individuals, supporting the specification of mixed-effect models to account for the nesting of observations within-persons.

Next, we specified a model containing the direct effects of our focal variables on engagement. As presented in Model 1 in Table 2, we found no significant effect of daily COVID-19 cases on daily engagement (*Coef*. = −0.102; *p* = 0.502). Yet, it is noteworthy that Model 1, including a random effect for COVID-19 cases, fitted notably better than a model with a fixed slope for daily COVID-19 cases (Δ AIC = 11.796; likelihood ratio = 13.80, *p* < 0.001). The improvement in model fit indicates that the effect of COVID-19 cases on engagement notably varies between observations, thereby pointing to the relevance of moderators of the relationship.

**Table 2:** Multilevel model predicting daily engagement.

	**Model 1**				**Model 2**				**Model 3**			
**Predictor**	***Coef*.**	***SE***	**95% CI**		***Coef*.**	***SE***	**95% CI**		***Coef*.**	***SE***	**95% CI**	
Intercept	3.597^**^	0.132	3.338	3.857	3.602^**^	0.132	3.343	3.861	3.601^**^	0.132	3.343	3.860
COVID	−0.102	0.151	−0.398	0.195	0.826	0.461	−0.077	1.728	0.869	0.474	−0.060	1.799
Age	0.008^**^	0.003	0.003	0.014	0.008^**^	0.003	0.003	0.013	0.008^**^	0.003	0.003	0.013
Leader consideration (CONS)	0.076^**^	0.013	0.051	0.102	0.077^**^	0.013	0.052	0.103	0.197^**^	0.052	0.096	0.298
COVID × Age					−0.021^*^	0.009	−0.039	−0.003	−0.022^*^	0.010	−0.041	−0.003
COVID × CONS					0.281^**^	0.087	0.110	0.451	−0.010	0.339	−0.675	0.655
Age × CONS									−0.003^*^	0.001	−0.005	−0.001
COVID × Age × CONS									0.006	0.008	−0.009	0.021
Time dummies	YES				YES				YES			
−2 log likelihood	5,651.386				5,640.402^**^				5,634.790			
AIC	5,681.385				5,674.402				5,672.791			

*N = 2,858 at the day-level and N = 388 at the person-level. Day-level predictors (COVID, CONS) were centered at each person's mean. COVID variable is rescaled (COVID Cases/1,000). Person-level predictor (Age) is uncentered. Time is dummy coded and modeled as fixed effect. Unstandardized effects are reported. Standard errors account for clustering at the regional level*.

* *p < 0.05 (two-tailed)*;

** *p < 0.01 (two-tailed)*.

**Figure 2:**
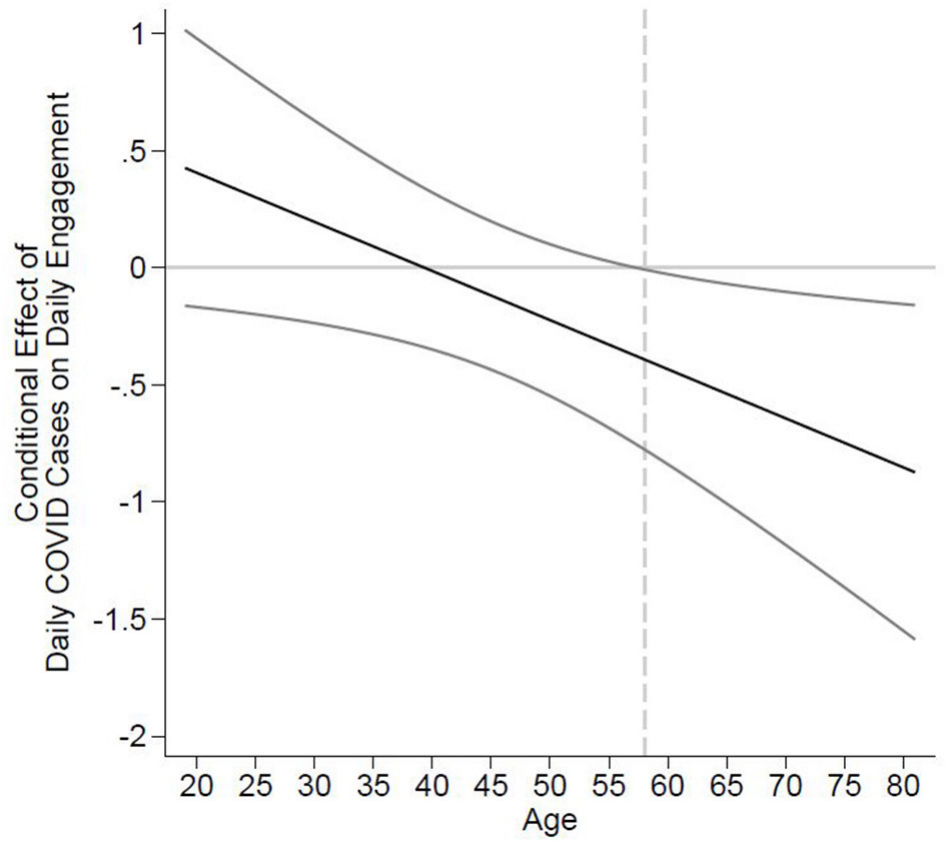
Johnson–Neyman regions of significance for the conditional relation between daily COVID-19 cases and daily engagement at realized values of chronological age. The black line depicts the conditional effect of daily COVID-19 cases on daily engagement (*y-*axis) dependent on the value of the age moderator (*x-*axis). The curved lines are the 95% confidence intervals of the conditional effect. The dashed vertical line represents the point at which the confidence interval does not include zero marking the region of significance of the conditional effect.

In the next step, we examined if the effect of daily COVID-19 cases in a county on daily engagement is more negative for older employees, as suggested in Hypothesis 1. The coefficient of the cross-level interaction between daily COVID-19 cases and chronological age in Model 2 is significant (*Coef*. = −0.021; *p* = 0.025). To facilitate the interpretation of the interaction, we followed recent recommendations and probed the interaction using the Johnson–Neyman technique (Preacher et al., 2006; Gardner et al., 2017). Compared with conventional simple slope tests at plus and minus one standard deviation of the moderator, the procedure allowed us to accurately determine the values of the age variable at which the effect of COVID-19 cases on daily engagement is (non-)significant. In support of Hypothesis 1, we found that daily local COVID-19 cases have a significant negative relation (*p* < 0.05) with next-day work engagement for employees older than 57.4 years, while the effect is not significant for employees younger than that. A plot of the conditional effect of COVID-19 cases on daily engagement (*y* axis) dependent on the age of the individual (*x* axis) is provided in Figure 2. Points for which the confidence intervals do not include zero indicate significant conditional effects.

**Figure 3:**
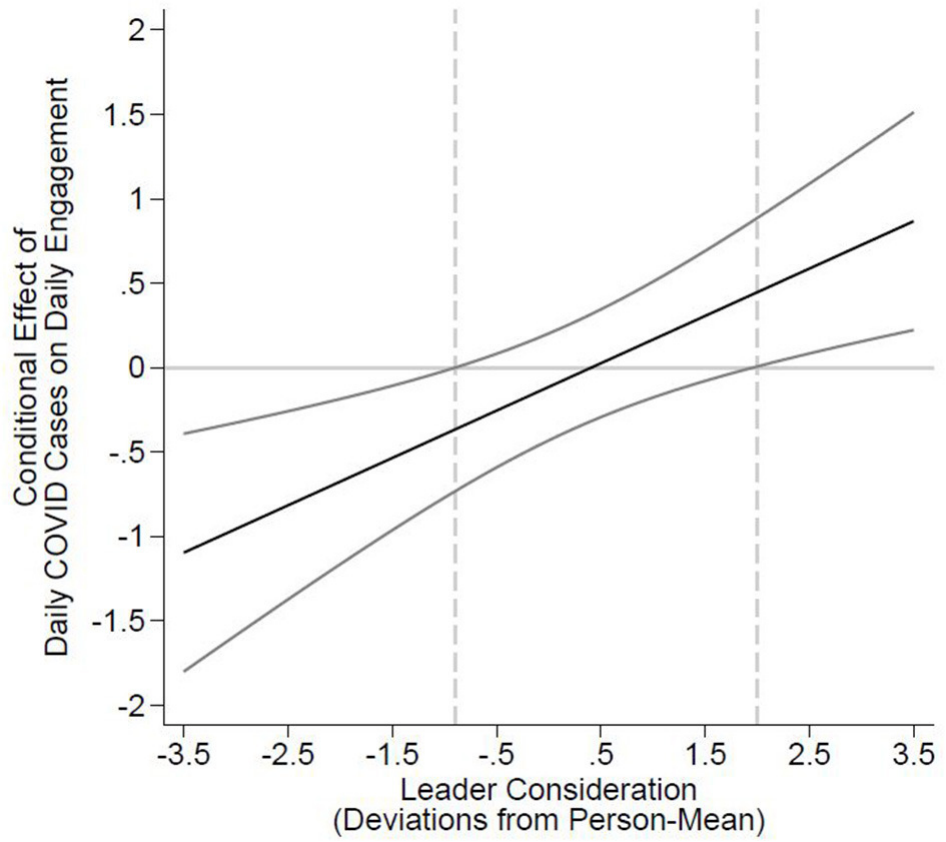
Johnson–Neyman regions of significance for the conditional relation between daily COVID-19 cases and daily engagement at realized values of leader consideration.

Next, we tested Hypothesis 2, which suggests that COVID-19 cases are more negatively related to employee daily engagement when employees receive low leader consideration. The two-way interaction between COVID-19 cases and leader consideration in Model 2 is significant (*Coef*. = 0.281; *p* = 0.001). By probing the interaction using the Johnson–Neyman technique, we find that COVID-19 cases are negatively related to daily engagement for individuals with leader consideration lower than −0.9 (person-mean centered) and are positively related when individuals receive leader consideration above 1.96 (when age is fixed to the sample mean). In our sample of 388 individuals, 187 (48.196%) report at least once a consideration value below −0.9 and thus experience the negative effect of local COVID-19 cases in their daily engagement. In contrast, only 65 (16.753%) individuals in our sample report leader consideration above 1.96 at least once and thus showed increased daily work engagement in response to rising local COVID-19 cases. Generally, the interaction pattern supports Hypothesis 2, and the corresponding plot is provided in Figure 3.

In the last step, we tested if older employees' engagement is most responsive to leader consideration in times of rising COVID-19 cases. As shown in Model 3 in Table 2, the three-way interaction among daily COVID-19 cases, age, and daily leader consideration is close to zero and not significant (*Coef*. = 0.006; *p* = 0.410). Thus, Hypothesis 3 is not supported.

## Discussion

Our analyses revealed that an external crisis event, such as the COVID-19 pandemic, has implications for work-related behavior within organizations. In line with the conceptual ideas of the event system theory (Morgeson et al., 2015), we could show that strong external events have the potential to spill over into the workplace by affecting employees' work engagement. Our study, therefore, corroborates an emerging stream of research (Lin et al., 2021; Liu et al., 2021), demonstrating the explanatory power of the events system theory to account for the influence of the COVID-19 pandemic on employee work behavior.

Furthermore, our study highlights the employees' age-based vulnerability and perceived leadership support to be decisive in determining whether the emerging COVID-19 pandemic is perceived as a strong event. Both factors might affect the sensemaking processes (Weick, 1988) in the pandemic and consequently, alter employment engagement levels. In detail, we could show that, over 8 days, daily engagement was only adversely affected by newly released local infection rates, if the employees are older than 57.4 years. This tipping point seems plausible, as the German Federal Ministry of health labels individuals 60 and older the main “Risk Group” in their official communication (German Federal Ministry of Health, 2020).

Furthermore, we could show that employees only lower their work engagement based on local increases in COVID-19 cases, if they receive low levels of personal consideration from their direct supervisor. This finding adds to the literature emphasizing the critical role of leadership in extreme or crisis contexts (Hannah et al., 2009). Even contrary to our expectations, high levels of perceived leadership consideration in times of surging infection rates can increase work enagement. Interestingly, the study by Hu et al. (2020) reveals similar findings indicating that the relationship between COVID-19 induced state anxiety and job engagement becomes positive when servant leadership is higher. We would speculate that these positive effects might be achieved by effective leaders who can use the extreme emotions released on the follower side during the pandemic (Hannah et al., 2009). These leaders might be able to transfer this high arousal via sensemaking strategies and cognitively shift the followers' perspective (Foldy et al., 2008) into positive engagement.

In contrast, we did not find that employees' age and received leader consideration interact with local infection rates such that older employees have the lowest engagement if they also receive low support from their supervisor. This finding indicates that leaders should not differentiate their individual consideration behavior between age groups in pandemic crises, which is in line with other results in the literature on differentiated leadership (Wu et al., 2010; Kunze et al., 2016). The open question, however, is how adverse effects of extern COVID-19 infections on older employees' engagement can be buffered. A promising avenue for future research might be to consider the conceptual literature that differentiates between different cognitive reflection processes that are triggered by mortality cues, such as a pandemic (Grant and Wade-Benzoni, 2009). More specifically, the core idea of this theory stream is that extreme external events cause both negative processes of death anxiety and more positive processes of death reflection. If supervisors can instill a somewhat positive process of death reflection instead of negative emotions and anxiety in their aging employees, they might be able to at least buffer the adverse effects of an external pandemic on work engagement.

Overall, our results have implications beyond the specific phenomenon of the COVID-19 pandemic for the emerging literature that links extreme external events to within-organizational processes and behavior (James, 2011; Hällgren et al., 2018). Our finding indicates that not only terrorist attacks and natural catastrophes but also health crises influence employee behavior. Furthermore, we introduce system event theory as a theoretical framework to explain such cross-level effects of disrupted environments on employee behavior.

### Practical Implications

Our results have implications for executives in the current pandemic situation and beyond. Based on empirical evidence from other pandemics, it is likely that the COVID-19 pandemic will continue with multiple infection peaks, with national and regional differences (Merler et al., 2008). Companies and respective leaders have to ensure that these pandemic waves do not adversely affect the engagement of their employees and followers. High employee engagement is crucial for general firm productivity, especially in the current pandemic crisis that challenges the profitability and even the existence of many companies.

Our findings imply that companies should be sensitive to the effects of rising COVID-19 infection rates for their elderly employees, although all age groups are potentially impacted by the pandemic (Adella Halim et al., 2020). Notably, in the case of a further intensive wave of the pandemic, companies should reduce work demands on this group of employees, as the extreme external context emotionally and cognitively strains them. Of course, we cannot fully rule out that a pandemic situation might also affect younger employees, especially if they worry about potential or actual infections of their older relatives or younger employees who have medical preconditions themselves putting them at higher personal risk for a severe course of the disease.

Additionally, our results reveal that a pandemic situation requires intensive leadership behaviors. If supervisors invest in individual consideration behaviors, they can prevent negative impacts of increasing COVID-19 cases on employee engagement and, in some cases, even enable higher engagement levels. In consequence, updated leadership training programs should sensitize executives for their responsibilities during extreme events, such as a pandemic.

### Limitations

Despite multiple strengths, such as repeated measurement design over eight workdays and daily COVID-19 cases as an exogenous independent variable, our research also has limitations that should be considered when interpreting the results. For example, our sample, also being representative in terms of age and gender for the German working population, was restricted to mainly white-collar workers with the potential to work remotely. In consequence, we would encourage future research to also extend this sample to blue-collar workers, who are even more at risk of catching the virus based on their physical presence at their workplace. We would expect that our observed effects of the virus vulnerability for aging employees would be even more severe in a sample of blue-collar employees working in face-to-face settings.

Additionally, our data collection took place in the first wave of the Covid-19 pandemic in spring 2020 in Germany, with relatively lower absolute infection rates than in subsequent waves. As such we would expect even stronger effects in later waves, with higher infection numbers, or in countries that were even harder affected by the Covid-19 pandemic. Thus, we recommend a replication of our findings in these settings.

Last, we focused only on age as one potential risk factor which may increase the severity of a COVID-19 and did not study other risk factors also prevalent among younger adults, such as diabetes. Based on our theoretical reasoning also younger adults with medical preconditions may reduce their engagement in response to rising local COVID-10 cases. We encourage future research to study this possibility.

### Conclusion

The COVID-19 pandemic is an unprecedented situation for employees, firms, and society as a whole. Our study is among the first to show that daily events of local infection numbers do affect the work engagement of employees within organizations. In particular, aging employees, who are most at risk of getting severe health issues from the virus, significantly lower their engagement in the case of surging infection rates. Additionally, we could show that individualized leadership behavior is effective in preventing adverse impacts of a pandemic on work engagement for all age groups. Beyond the current pandemic, the results also have broader implications for the literature that links extreme external incidents with organizational behavior (Hällgren et al., 2018).

## Data Availability Statement

The raw data supporting the conclusions of this article will be made available by the authors, without undue reservation.

## Ethics Statement

Ethical approval was not provided for this study on human participants because for field survey studies the University of Konstanz does not require an ethical approval. The patients/participants provided their written informed consent to participate in this study.

## Author Contributions

MR did the analyses and wrote parts of the paper. SZ and FK conceptualized the study and wrote parts of the paper. All authors contributed equally to this study and jointly developed the initial idea.

## Conflict of Interest

The authors declare that the research was conducted in the absence of any commercial or financial relationships that could be construed as a potential conflict of interest.
